# Athlete data sovereignty: addressing the legal and policy gaps in sports technology

**DOI:** 10.3389/fspor.2025.1742484

**Published:** 2025-12-15

**Authors:** Jun Woo Kwon

**Affiliations:** Sports Technology Laboratory, Department of Physical Education, Seoul National University, Seoul, Republic of Korea

**Keywords:** athlete data sovereignty, co-ownership, general data protection regulation (GDPR), governance, personal information protection act (PIPA), privacy law, sports technology

## 1 Introduction

Sports technology has rapidly reshaped the measurement and governance of athletic performance. Wearables, smart fabrics, and AI-linked sensors now generate constant biometric and tactical streams used for analytics, injury prevention, and fan-facing applications. As these tools become embedded in everyday training, the line between assistance and surveillance becomes harder to distinguish, raising new concerns about autonomy and control (1). Although the data originate from athletes’ bodies, legal ownership remains uncertain (2). Existing privacy law and international sports governance frameworks offer no clear allocation of rights over the digital traces produced through play or training (3). The concept of data sovereignty-first developed in Indigenous data governance and later expanded into digital rights debates-suggests that individuals should retain authority over information tied to their bodily identity (4). Yet, despite its relevance, this principle has rarely been applied to the sporting context (5). This absence of legal clarity creates a paradox. Athletes generate the data that underpin modern sports technology, yet remain largely excluded from decision-making about its use. Without a recognised ownership framework, performance data sit in an ambiguous space: deeply personal yet institutionally controlled, private yet commercially valuable (6).

## 2 Definitions

To avoid uncertainty in interpretation, several key terms are defined at the outset. First, *performance data* refers to biometric or sport-generated information produced during training or competition. This category includes measurable signals such as heart-rate output, GPS tracking traces, neuromuscular load, movement efficiency markers, reaction-time scores, and other analytics derived directly from an athlete's body or actions. Unlike ordinary personal data collected outside a workplace, these data exist only because the athlete performs. Second, the term *data sovereignty* is used here to describe an individual's authority to decide how information linked to their physical identity is collected, accessed, shared, stored, and potentially erased. The idea originally emerged in Indigenous data governance debates, but it now informs wider discussions about digital autonomy and informational rights. Finally, *co-ownership* is not presented as a settled legal rule but as an emerging governance model relevant to sport. Under this model, data rights and responsibilities are shared among athletes, clubs, leagues, and technology providers. Rather than permitting unilateral control by employers or vendors, co-ownership assumes that access, secondary use, retention, and commercial exploitation must be negotiated and justified.

## 3 The unclear ownership of athlete data

Athlete performance data differ from ordinary personal information because they are generated through professional activity and combine physiological, biomechanical, and tactical elements (1). Their production is inseparable from physical labour and competitive action, meaning they exist only because the athlete performs. This hybrid character-both biological and professional-complicates the question of who holds legitimate control. Despite this, prevailing regulatory and contractual structures treat such information as employer-managed output rather than an extension of personhood (3). Many leagues include provisions permitting extensive use of biometric or performance data for analytics, marketing, or third-party licensing (3). In these contexts, consent is typically formal rather than substantive due to structural bargaining inequality between athletes and organisations (5). Over time, this asymmetry has normalised treatment of athlete-generated data as proprietary institutional assets rather than material tied to identity.

Although the GDPR recognises individual rights including access (Art. 15), correction (Art. 16), erasure (Art. 17), restriction (Art. 18), portability (Art. 20), and objection (Art. 21), supported by transparency duties under Articles 12–14, exercising these rights in elite sport remains challenging. Once biometric records enter layered analytic ecosystems, athletes encounter fragmented storage, opaque processing, and procedural barriers, rendering legal rights difficult to operationalise (7). Institutional platforms often integrate medical, performance, and commercial modules, making data lineage unclear and deletion or correction technically difficult. Although bodies such as the IOC and FIFA provide guidance on secure handling, they refrain from addressing ownership or benefit-sharing (6). As a result, athletes often do not know how long their information is retained, whether it is monetised, or who profits from derivative datasets (2). What begins as performance optimisation can therefore evolve into an opaque economic system that privileges institutional control.

This unresolved framework creates a conceptual gap: athlete data are simultaneously personal, professional, and commercial, yet legal regimes force a binary classification (1). The result is an athlete positioned primarily as a passive data subject rather than a rights-bearing contributor (4). Real-world disputes demonstrate that this ambiguity is not theoretical. NBA players have questioned whether teams may claim rights over analytic outputs derived from wearables even where collection was negotiated through union channels (8). Comparative labour analysis similarly shows that although leagues such as the NFL and MLB reference wearable technology, most agreements fail to define ownership, access conditions, or benefit-sharing obligations (9).

## 4 Legal and policy vacuum

Korea's Personal Information Protection Act (PIPA) focuses on controller duties but does not recognise any proprietary claim athletes may hold over data produced from their bodies (5). Although it regulates consent, purpose limitation, and retention, it frames personal information as institution-managed rather than individually controlled. For athletes, this offers procedural compliance but no substantive right acknowledging that such data are inseparable from identity and livelihood. Consent remains the default legal basis, yet in elite sport voluntariness is largely symbolic (3). Employment dependency and contractual imbalance mean refusal is rarely realistic (1). Thus, even where consent appears valid, it often functions as a condition of participation rather than genuine self-determination, reinforcing institutional dominance rather than protecting autonomy.

Similar tensions appear internationally. In Australia, recent debate over biometric tracking highlights both “consent fatigue” and uncertainty surrounding retention and secondary uses (6). Athletes may know their data are being collected but lack clarity on how long they are stored, how algorithms evaluate them, or whether they may resurface in commercial or evaluative contexts-issues that carry career-long consequences. While discussions of data sovereignty are emerging in the EU and Australia, they seldom extend to sport (4). This omission leaves athletes largely invisible within digital rights policy, despite being among the most continuously monitored populations. As technology vendors increasingly control analytics and storage, athletes become sources of value without corresponding control or benefit-sharing (2, 6). In such an environment, biometric information risks becoming a commodified asset rather than protected personal data.

Given that performance metrics may reveal medical, psychological, or fatigue-related indicators, athlete data arguably require protection comparable to medical information (3). Yet existing privacy law rarely distinguishes performance metrics from routine employment records or mandates heightened safeguards (5). This gap reduces the athlete to an administrative entry rather than a rights-bearing individual.

These unresolved questions echo broader theoretical debates. The German Constitutional Court's Volkszählungsurteil tied data control to personal liberty and the ability to determine one's informational boundaries (10). Radin similarly argued that certain information merges with identity such that losing control undermines dignity (11). Posthuman scholarship extends this logic, suggesting that sensor-enhanced athletes form continuous systems of body and data rather than two separable entities (12). From this standpoint, athlete data resemble an extension of personhood rather than a work-product. Accordingly, the core question-“Who owns the athlete's data?”-remains unresolved (2). This absence of clarity weakens trust, creates legal uncertainty, and chills ethical technological development (6).

However, governance is not entirely absent. In some jurisdictions, labour negotiation rather than legislation is beginning to structure rights. Article 55 of the 2020 NFL–NFLPA agreement requires consent, limits secondary uses, and introduces joint oversight (13). The NBA's 2023 agreement adopts a similar model, granting athletes access rights and prohibiting commercial use without renewed approval (14). These negotiated frameworks signal the emergence of shared governance and provide early templates for future models based on co-ownership and athlete data sovereignty.

## 5 Toward an athlete-centric data governance

Bridging these gaps requires reframing athlete data governance through three interlocking reforms (4, 6). Such a framework must recognise that performance data are lived expressions of athletic labour rather than neutral technical outputs. Accordingly, governance should follow principles applied to other sensitive human data-transparency, proportionality, and respect for dignity-while moving beyond compliance and toward genuine participation in decisions about collection and use. Although no legal system currently recognises athlete performance data as jointly owned property, several developing legal concepts indicate possible future directions. Within the EU, for instance, case law on joint controllership shows that more than one entity may share decision-making authority over the same set of data (15, 16). Outside the courtroom, professional sport already shows early movement in this direction. Collective bargaining agreements in leagues such as the NFL and NBA function less as unilateral employer control and more as negotiated frameworks of shared governance (13, 14). Additionally, arguments rooted in theories of identity and personhood suggest that athlete-generated information may deserve treatment closer to rights inherent to the individual, rather than being reduced to a transferable commercial asset (10, 11).

First, a co-ownership model could be adopted to allocate shared rights among athletes, clubs, and technology providers (2, 3). While not yet formalised in sport, the idea aligns with legal developments. EU jurisprudence-including Wirtschaftsakademie (Case C-210/16) and Fashion ID (Case C-40/17)-confirms that control over the same dataset may be shared under the GDPR's doctrine of joint controllership (15, 16). A similar trend is emerging in practice: under the 2020 NFL–NFLPA collective bargaining agreement, biometric-tracking decisions are jointly administered rather than imposed unilaterally. Treating data as a shared resource rather than an employment artefact would help prevent exclusive institutional control and enable negotiated limits on secondary use, commercialisation, and retention (5). Second, data sovereignty should be expressly integrated into sport-governance instruments (4). Instead of being treated merely as data subjects, athletes should be recognised as legitimate data owners with authority to approve, monitor, and revoke processing (1, 6). Embedding sovereignty into league rules and federation policy would shift athletes from passive compliance to active participation and align sport governance with broader principles of autonomy and human-rights-based data protection. Third, federations and national associations should establish enforceable ethical and legal guidelines defining permissible collection, retention periods, and commercial boundaries (5). Tools such as standardised consent workflows, transparency audits, and independent oversight boards would help translate principles into practice and prevent nominal compliance becoming symbolic (3).

Taken together, these measures support a more legitimate and equitable digital sport ecosystem. Recognising athlete data sovereignty not only protects privacy but also reshapes fairness in technology-mediated competition (4, 5). Respecting informational autonomy is not a barrier to innovation-it is a necessary condition for a sustainable, trusted, and ethically credible sports-technology environment (1).

## 6 Summary

Overall, this commentary has shown that athlete performance data should not be treated as ordinary workplace information. These data are deeply tied to identity and bodily integrity, and current legal frameworks have not kept pace with the realities of sensor-based sport. The analysis highlights that fragmented regulation leaves athletes with rights in theory but limited authority in practice. Yet, emerging governance models-most visibly in collective bargaining and joint controllership jurisprudence-suggest that change is already underway. Building on these developments, future reforms should recognise athlete data sovereignty, establish shared governance mechanisms, and ensure independent oversight capable of addressing commercial and technological pressures. The aim is not merely regulatory tidiness; it is to protect fairness, autonomy, and trust in an increasingly data-mediated sporting environment. While this article does not claim to offer a complete regulatory blueprint, it provides direction and a conceptual basis for further policy, legal, and scholarly development.
